# The ICU-Diary study: prospective, multicenter comparative study of the impact of an ICU diary on the wellbeing of patients and families in French ICUs

**DOI:** 10.1186/s13063-017-2283-y

**Published:** 2017-11-15

**Authors:** Maïté Garrouste-Orgeas, Cécile Flahault, Léonor Fasse, Stéphane Ruckly, Nora Amdjar-Badidi, Laurent Argaud, Julio Badie, Amélie Bazire, Naike Bige, Eric Boulet, Lila Bouadma, Cédric Bretonnière, Bernard Floccard, Alain Gaffinel, Xavier de Forceville, Hubert Grand, Rebecca Halidfar, Olfa Hamzaoui, Mercé Jourdain, Paul-Henri Jost, Eric Kipnis, Audrey Large, Alexandre Lautrette, Olivier Lesieur, Virginie Maxime, Emmanuelle Mercier, Jean Paul Mira, Yannick Monseau, Erika Parmentier-Decrucq, Jean-Philippe Rigaud, Antoine Rouget, François Santoli, Georges Simon, Fabienne Tamion, Nathalie Thieulot-Rolin, Marina Thirion, Sandrine Valade, Isabelle Vinatier, Christel Vioulac, Sebastien Bailly, Jean-François Timsit

**Affiliations:** 1 Infection, Antimicrobials, Modelling, Evolution (IAME), UMR 1137, INSERM and Paris Diderot University, Department of Biostatistics − HUPNVS − AP-HP, UFR de Médecine − Bichat University Hospital, Paris, France; 2 Department of Biostatistics, Outcomerea, Paris, France; 3 Medical unit, French British Hospital Institute, Levallois-Perret, France; 4 Psychology laboratory and work process, Paris Descartes University, Paris, France; 5 Laboratoire Psy-DREPI EA-7458, Bourgogne Franche Comté University, Dijon, France; 6 Medical-Surgical ICU, General Hospital René Dubos, Pontoise, France; 7 Medical ICU, Edouard Herriot University Hospital, Lyon, France; 8 Medical-Surgical ICU, General Hospital Belfort-Montbeliard, Belfort, France; 9 Medical ICU, La Cavale Blanche University Hospital, Brest, France; 10 Medical ICU, Saint Antoine University Hospital, Paris, France; 11 Medical ICU, Beaumont General Hospital, Beaumont, France; 12 Medical ICU, Bichat University Hospital, Paris, France; 13 Medical ICU, Nantes University Hospital, Nantes, France; 14 EA3826, Laboratory of clinical and experimental therapeutics of infections, University of Nantes, Nantes, France; 15 Medical ICU, Hospices Civils de Lyon, Edouard Herriot University Hospital, Lyon, France; 16 Medical-Surgical ICU, Gustave Roussy Cancer Campus, Villejuif, France; 17 Medical-Surgical ICU, Est Francilien Hospital network, Meaux, France; 18 Medical-Surgical ICU, Hospital Robert Boulin, Libourne, France; 19 Medical ICU, Albert Michallon University Hospital, Grenoble, France; 20 Medical ICU, University Hospital Paris-Sud, Beclère University Hospital, Clamart, France; 21 Lille University, Inserm U1190, Lille, France; 22 Group of medical ICUs, Lille University Hospital, Lille, France; 23 Surgical ICU, Henri Mondor University Hospital, Créteil, France; 24 Surgical ICU, Lille University Hospital, Lille, France; 25 Medical ICU, François Mitterrand University Hospital, Dijon, France; 26 Medical ICU, Gabriel-Montpied University Hospital, Clermont Ferrand, France; 27 LMGE UMR CNRS 6023, University of Clermont-Ferrand, Clermont Ferrand, France; 28 Medical-Surgical ICU, General Hospital, La Rochelle, France; 29 EA 4569, University Paris Descartes, Paris, France; 30 Medical ICU, Raymond Poincaré University Hospital, Garches, France; 31 CRICS group, Medical-Surgical ICU, Tours University Hospital, Tours, France; 32 Medical ICU, Cochin University Hospital, Paris, France; 33 Medical-Surgical ICU, General Hospital, Périgueux, France; 34 Department of Intensive Care, Dieppe General Hôpital, Dieppe, France; 35 Medical-Surgical ICU, Rangueil University Hospital, Toulouse, France; 36 Medical ICU, General Hospital Robert Ballanger, Aulnay-Sous-Bois, France; 37 Medical-Surgical ICU, General Hospital, Troyes, France; 38 Medical ICU, University medical center, Rouen, France; 39 INSERM U-1096, University of Rouen, Rouen, France; 40 Medical-Surgical ICU, General Hôpital, Melun, France; 41 Medical-Surgical ICU, General Hospital Victor Dupouy, Argenteuil, France; 42 Medical ICU, Saint Louis University Hospital, Paris, France; 43 Medical ICU, Les Oudaries Hospital, La Roche-sur-Yon, France

**Keywords:** ICU diary, Intensive care unit, Stress disorders, post-traumatic, Family, Anxiety, Depression

## Abstract

**Background:**

Post-intensive care syndrome includes the multiple consequences of an intensive care unit (ICU) stay for patients and families. It has become a new challenge for intensivists. Prevention programs have been disappointing, except for ICU diaries, which report the patient’s story in the ICU. However, the effectiveness of ICU diaries for patients and families is still controversial, as the interpretation of the results of previous studies was open to criticism hampering an expanded use of the diary. The primary objective of the study is to evaluate the post-traumatic stress syndrome in patients. The secondary objectives are to evaluate the post-traumatic stress syndrome in families, anxiety and depression symptoms in patients and families, and the recollected memories of patients. Endpoints will be evaluated 3 months after ICU discharge or death.

**Methods:**

A prospective, multicenter, randomized, assessor-blind comparative study of the effect of an ICU diary on patients and families. We will compare two groups: one group with an ICU diary written by staff and family and given to the patient at ICU discharge or to the family in case of death, and a control group without any ICU diary. Each of the 35 participating centers will include 20 patients having at least one family member who will likely visit the patient during their ICU stay. Patients must be ventilated within 48 h after ICU admission and not have any previous chronic neurologic or acute condition responsible for cognitive impairments that would hamper their participation in a phone interview. Three months after ICU discharge or death of the patient, a psychologist will contact the patient and family by phone. Post-traumatic stress syndrome will be evaluated using the Impact of Events Scale-Revised questionnaire, anxiety and depression symptoms using the Hospital Anxiety and Depression Scale questionnaire, both in patients and families, and memory recollection using the ICU Memory Tool Questionnaire in patients. The content of a randomized sample of diaries of each center will be analyzed using a grid. An interview of the patients in the intervention arm will be conducted 6 months after ICU discharge to analyze in depth how they use the diary.

**Discussion:**

This study will provide new insights on the impact of ICU diaries on post-traumatic stress disorders in patients and families after an ICU stay.

**Trial registration:**

ClinicalTrial.gov, ID: NCT02519725. Registered on 13 July 2015.

**Electronic supplementary material:**

The online version of this article (doi:10.1186/s13063-017-2283-y) contains supplementary material, which is available to authorized users.

## Background

Hospitalization in an intensive care unit (ICU) is a stressful experience for both patients and families, as they are facing an unknown environment, diagnostic and prognostic uncertainty, and possible fears of dying and of having an impaired quality of life. The progression of intensive care therapies is associated with decrease of in-ICU and in-hospital mortality [1–4]. However, this reduction in mortality is not the main criteria for the patients’ and families’ evaluation of their ICU stay, as the wishes of most survivors are to regain their personal and professional lives [5–7].

For several years, attention has been focused on the ICU being the origin of patients’ and family’s sequelae [8–10]. In 2012, a conference of stakeholders representing key organizations and groups of North America described the “post-intensive care syndrome” (PICS) as including physical, cognitive and psychological consequences of ICU stay for patients (PICS) and families (PICS-F) [11, 12]. Although there is a lack of basic scientific evidence enabling the understanding of the pathological mechanisms of post-ICU morbidity, experts agree on the importance of ICU events (hypoxia, shock, inflammation, glucose dysregulation), ICU treatments (use of benzodiazepines and other sedatives), and ICU conditions (bed immobilization, physical restraint, discomforts like noise, pain, thirst) [13]. Cognitive impairments can persist in 30 to 80% of patients. More than 6 months after hospital discharge, anxiety, depression, and post-traumatic stress-related syndrome (PTSD) are described in 8–57%, 23–48%, and 10–50% of patients, respectively [14]. Herridge et al. described four post-ICU disability stages based on age, duration of ICU stay and physical functional dependency 1 week after ICU admission, without significant differences in cognitive status [15]. At 6 months, anxiety, depression symptoms, and PTSD were described in 15–24%, 5–36%, and 35–57% of the families, respectively [16]. To cope with these syndromes, 8 to 32% of families used medications after ICU admission; the ICU stay interfered with their lifestyle in half of them while they were caring of their relatives [16]. Most of ICU survivors required relative’s assistance after their ICU stay, adding additional burden to their own life, especially depressive symptoms for up to 1 year [17]. These findings suggest that critical illness affects long-term outcome not only in patients but also in their relatives, increasing the effect of critical illness and societal burden. Some experts spoke about the “hidden public health disaster” [18]. Prevention programs are needed to limit these consequences.

Several prevention programs have focused on psychological consequences, and results were often disappointing [19–26]. Three types of prevention programs were evaluated: early in-ICU psychological interventions, post-ICU coping skill interventions through phone visits for outpatients, and ICU diaries. In a single, before-after study, the intervention of a psychologist for trauma patients providing stress support and coping strategies for management of anxiety, depression, PTSD, fear, and discomfort yielded a significant decrease in the PTSD syndrome [26]. Jones et al. included ventilated patients in a randomized controlled trial with blinded follow-up that evaluated an intervention associating a manual with recommendations of physical exercises, psychosocial and psychological advices, and thrice weekly phone calls reinforcing the use of the manual. This intervention showed conflicting results, with no effect on anxiety, depression or PTSD, and a significant increase of quality of life up to 6 months [19]. In a single, small, randomized controlled study of 21 survivors, Jackson et al. showed that a cognitive, physical, and functional rehabilitation had an effect in improving cognitive performance [24]. As the studies are scarce and limited, the level of evidence for promoting these interventions is currently weak.

One of the explanations for the emotional troubles experienced by these patients is the lack of memories or the presence of delusional memories of their ICU stay [27, 28]. The intervention tested to fill the memory gap has been the ICU diary [29]. The ICU diary has been used for 30 years in North Europe and further extended to other European countries, North America, and Australia [30–32]. The diary is prospectively written in an everyday language and builds the patient’s story. It contains the reason of admission, a daily narrative of activity, and a conclusion before transferring the patient to the ward, or condolence for the family for the decedents. Every entry is signed and the diary may contain different numbers of entries. Entries are mainly made by the bedside nurse; families are encouraged to write in it; in some countries, physicians also participate to construct the patient’s story [22, 31, 33, 34]. Several studies explored the influence of the diary on the psychological consequences of the ICU stay on patients and families. A European randomized study included 372 patients with an ICU stay of more than 72 h, where half of them received the diary 1 month after ICU discharge. The onset of PTSD was reduced from 13 to 5% in the group of patients having received the diary [35]. A French single-center study including patients with an ICU stay of more than 96 h showed that the ICU diary was associated with a decrease of PTSD syndrome in patients and families 1 year after ICU discharge [22]. Some recent reports reviewed the impact of diaries on patient’s recovery and yielded conflicting results due to methodological features [36–38]. Studies of impact were single-center studies [20, 22], with different patient case-mix [22, 39], and those where diaries were written by different categories of caregivers. The evaluation of the impact, and the methodology used (qualitative or quantitative) differed between studies [21, 40, 41]. These differences did not recommend the use of a diary in routine clinical practice.

These findings prompted us to design the ICU-Diary study to overcome these limitations and clarify the impact of an ICU diary on patients and families.

## Methods

### Study design

**Fig. 1:**
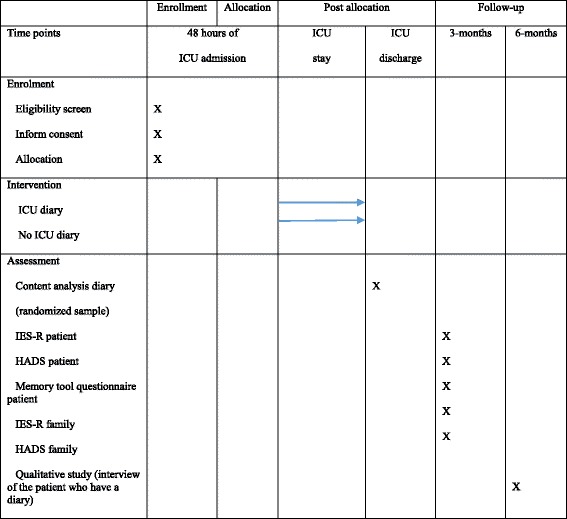
Study timeline, schedule of enrollment, interventions, and assessments

The ICU-Diary study is a multicenter, randomized, parallel (allocation ratio 1:1), and controlled study to assess the impact of the ICU diary on the wellbeing of both patients and families. We designed a quantitative study to assess the psychological effect of the diary and a qualitative study to examine how the patient used his diary 6 months after ICU discharge. The study was not blinded for the ICU staff but assessment was blinded for the psychologist processing the follow-up. The study timeline and schedule of enrollment, interventions, and assessments is presented in Fig. 1. The Standard Protocol Items: Recommendations for Interventional Trials (SPIRIT) Checklist is provided in Additional file 1.

### Aims

The primary objective is to evaluate the PTSD in patients 3 months after ICU discharge as measured by the Impact Event Scale-Revised questionnaire (IES-R). The IES-R is a validated and reliable tool for the evaluation of PTSD symptoms of intrusion, avoidance, and hyperarousal [42]. Each item is scored on a 5-point Likert-type scale (0, not at all; 1, rarely; 2, occasionally, 3, fairly often; and 4, often). The total score can range from 0 (no symptoms) to 88 (severe symptoms). The main criteria of measurement will be the score of PTSD as having a a low or high IES-R score, using 22 as the cutoff, as previously used in ICU studies [43].

The secondary objectives are to evaluate:

- The PTSD in families 3 months after ICU discharge, using the IES-R scale [42]. The criteria of measurement will be the score of PTSD as having a low or high IES-R score, using 22 as the cutoff, as previously used in ICU studies [43]
- Anxiety and depression symptoms in patients 3 months after ICU discharge
- Anxiety and depression symptoms families 3 months after ICU discharge. Symptoms of anxiety and depression are assessed using the validated French version of the self-administered Hospital Anxiety and Depression Scale (HADS), which has seven items on anxiety and seven on depression [44]. The sum for each domain ranges from 0 to 21. A score at 7 or below is considered normal. The cutoff score of 8 for each of the two subscales defines severe symptoms of anxiety or depression [45]
- The recollection of memories of the ICU stay by the patient 3 months after ICU discharge, as measured by the ICU Memory Tool Questionnaire [46]. The ICU Memory Tool Questionnaire consists of items exploring recollections of the patient before and during their ICU stay. It also includes two questions to assess if PTSD symptoms are present. As no French version exists, the ICU memory tool questionnaire was translated and back-translated into French by bilingual researchers, namely a French native who speaks English and an English native who speaks French
- Diary content analysis
  The content of a random sample of the study diaries will be analyzed with a grid (Table 1) that has already been published [22]. This grid was built with a panel comprising 11 members (three ICU physicians including two from other units, one ICU nurse, two psychologists, two hospital visiting volunteers, one person from the general population with a history of admission to another ICU, one former patient of our ICU, and his wife) using a Delphi technique. Six categories were defined and will serve for the analysis of the content of the diaries. We will report quantitatively the categories and themes which are represented most often in the diaries and will give verbatim examples for each of them.

**Table 1:** Grid of analysis of the content of the intensive care unit (ICU) diary

Category 1: Defining places, spaces, and people	
Theme 1	Narrative about the ICU and its location in the hospital and city
Theme 2	Narrative about the identity and job responsibilities of each ICU staff member
Theme 3	Narrative about the characteristics of the room
Theme 4	Narrative describing the pictures posted in the room
Theme 5	Narrative about the characteristics and purpose of the machines
Theme 6	Narrative about the sights and sounds in the room
Theme 7	Narrative about the presence of, or visits by, members of the clergy
Category 2: Building a time flow of medical events	
Theme 1	Narrative about the patient’s history before ICU admission and after the first urgent interventions
Theme 2	Narrative about the condition of the patient, clinical course, treatments, procedures, investigations, and surgeries
Theme 3	Description of events that interfered or might have interfered with the presence of ICU staff members at the bedside
Theme 4	Comments on life expectancy and the expected impact of the disorders on quality of life
Theme 5	Narrative describing exchanges among healthcare professionals involved with the patient: date of onset and content
Theme 6	Narrative about the differences in tasks carried out by the day staff and night staff, to explain how the 24-h cycle unfolds
Category 3: To replace the time flow of the patient’s experience within the time flow of family, community, and world events	
Theme 1	Narrative about events in the patient’s personal life (narrator, family, friends …)
Theme 2	Narrative about the difference in the perceived time flow of events between the patient since ICU admission and the narrator or family/friends
Theme 3	Narrative about future projects for the patient
Theme 4	Narrative about concomitant social, political, economic, and cultural events
Theme 5	Narrative about the visits, their sequence in time, their duration, and factors that prevented some visits from occurring
Category 4: To demonstrate the continuity of the patient’s life	
Theme 1	Narrative about the patient’s recent or remote past, habits, reactions, and personality features
Theme 2	Narrative about the patient’s behaviors, attitudes, and actions
Theme 3	Narrative about physical changes and attitudes (e.g., ability to open/close the eyes)
Theme 4	Narrative about changes in expressions of pain and responses to nursing care
Theme 5	Narrative about the patient’s emotional responses to the voices of the staff and family/friends (smiling, small movements of the eyelids or body)
Theme 6	Narrative about the patient’s emotional responses to physical contact (stroking, holding hands, touching …)
Category 5: To express feelings and emotions	
Theme 1	Narrative that explicitly describes feelings or emotions about the patient
Theme 2	Narrative that explicitly describes feelings or emotions about or toward the ICU staff
Theme 3	Narrative that explicitly describes feelings or emotions of family members or other loved ones
Theme 4	Narrative describing expectations, fears, discouragement, and hopes of the family and other loved ones
Theme 5	Narrative describing the fears and hopes of the staff
Category 6: To explicitly demonstrate the presence, commitment, and support of staff and family	
Theme 1	To write an account of one’s presence at the patient’s bedside
Theme 2	To make one’s presence felt in a personal and original way (poems, songs, music, drawings …)
Theme 3	To describe relationships between the patient and other persons while encouraging the other persons to speak to, and to touch, the patient, despite the unfavorable environment
Theme 4	To describe one’s physical involvement in communicating with the patient
Theme 5	To describe or refer to one’s support in the form of prayers or any other religious or spiritual activity
- The way the patient uses the diary will be explored through a qualitative approach, analyzing semidirective interviews with the patients of the intervention arm, performed 6 months after the ICU discharge using thematic and interpretative phenomenological analysis.

Primary and secondary objectives are measured by a psychologist hired for the study. The psychologist will be blind to the randomization arm when they will phone patients and families. If patients or a family member speak about the diary during the interview, the number of unblinded interviews will be reported in the study flow chart.

### Participants

#### Recruitment of ICUs

The participating ICUs belong to the French Society of Critical Care (SRLF) or the French Society of Anesthesiology (SFAR). The selection criteria were:

- Adult, medical, surgical or medical-surgical ICU patients
- Agreement of the whole team to write in the diaries
- Supervision of the study by physicians or nurses
- Agreement with the randomized design in the ICUs which previously used diaries

The participating ICUs are displayed in Additional file 2.

#### Recruitment of patients

Inclusion criteria are as follows:

- Age 18 years or older
- Mechanical ventilation for more than 48 h and initiated within the first 48 h after ICU admission
- Patient having a family member speaking and understanding French, and with the ability to visit them during the ICU stay
- Subject consenting to participate in the study, or obtention of family consent in case of patient incompetency

Non-inclusion criteria are as follows:

- Patient without family available
- Patient or family not speaking or understanding French
- Current or previously neurologic conditions that would preclude questionnaire completion such as pre-existing psychotic or dementia-type illness, hospitalization after a cardiac arrest, acute neurologic diseases (meningitis, ischemic or hemorragic stroke), or cerebral trauma patients
- Patient status considered by the investigator to be inevitably leading to death or to withdrawal of life support within 48 h
- Patient already included in a study with interview follow-up
- Patient deprived of liberty
- Deaf and mute patients

### Intervention

The intervention is the elaboration of an ICU diary by caregivers and families. All the diaries for every center are built on the same way by the main investigator and given to the different centers before the beginning of the study. The first page is standardized and includes an explanation of the purpose of the diary; then the diary includes a picture of a typical patient’s room with an explanation of the different devices possibly used during the stay, pictures of the unit, and a list of the main responsible persons of the unit. The diary is maintained by the family and by the ICU staff. The only instruction given to the families and staff members about diary entries is to refrain from writing about confidential matters that could not be shared among the patient, all relatives, and the staff. Staff members are free to express compassion and their hope, or absence thereof, that the patient would recover. Relatives can speak freely with the patients without guidance from the ICU staff. The first entry is written immediately after randomization by the ICU physician and includes a summary of the reason for admission, the name of the ICU and of the hospital, and a summary of the events of the first 48 h. Then, each day, a brief entry is mandatory during the whole ICU stay. This entry can be done by an ICU staff member whatever their category. Relatives are not encouraged to take photos of the patient during the whole stay without the patient’s permission. No patient’s photos taken during the ICU stay are allowed in the ICU diary, only pictures taken prior to the ICU stay (which were taken with the patient’s authorization). For patients in the intervention arm, they received the ICU diary at the conclusion of their ICU stay when they are discharged to wards. If the patient dies, the diary is given to the family if they are present in the unit or sent by mail with an identical letter for all ICUs. A guide for the elaboration of the ICU diary was given to each center containing: rules for writing in the diary, indications for opening and maintaining the diary, for writing that the patient status does not fit well when the clinical patient status impaired, and for relinquishing the diary at ICU discharge. Examples of verbatim narratives of different categories of healthcare workers (ICU physician, resident, nurse, nursing assistant, physiotherapist) are included in the guide. World Health Organization Trial registration Data Set is provided in Additional file 3.

### Training

A training on the study will be performed for each center by the main investigator at the study initiation visit. A meeting is planned, enabling the supervisors of the study to discuss the training with the ICU staff. The study will be presented and the guide of how to fill in the diaries will be discussed. Examples of verbatim narratives will be provided. A free discussion will be organized to address all potential difficulties for the ICU staff. The phone numbers of the main investigator (MGO) and of the two clinical researchers will be provided to each center for managing issues during the inclusion period. During the study period, the local co-investigator in each center is responsible for the supervision and promotion of the study, and supports the ICU staff for all potential difficulties for writing in the diaries.

### Arms

#### Intervention arm

An ICU diary is opened by the ICU staff for the patient and their relatives at 48 h after ICU admission. It will be given to the patient at ICU discharge or to his family in case of death.

#### Control arm

No diary is commenced during the ICU stay.

### Randomization

The 1:1 randomization is stratified by center and balanced with permutation blocks. Randomization is performed via a secure and dedicated website managed by the ICUREsearch company. Family members or patients are randomized at 48 h after ICU admission if they fulfill all inclusion and non-inclusion criteria.

### Data collection

#### ICU characteristics

The following ICU characteristics are recorded: hospital (university, community or private hospital), ICU (medical, surgical or medical-surgical), number of beds, structure of the unit (number of senior physicians and resident, nurse-to-patient ratio, nursing assistant-to-patient ratio).

#### Patient characteristics

The following patient characteristics are recorded: demographics (sex, age); health status before ICU admission (Knaus [47] and McCabe [48] classification), health status at ICU admission using the Simplified Acute Physiology Score [49], type of admission (medical, scheduled surgical, or emergency surgical); use and duration in days of mechanical ventilation, of non-invasive ventilation, sedation duration, iatrogenic events, length of ICU stay. The date and limitation of treatments are also collected.

#### Family characteristics

The following family characteristics are recorded: age, gender, relationship with the patient, educational level, professional status.

### Sample size justification

Post-traumatic stress-related syndrome was reported in 15% [35], 20.7% [50], 51% [19], and 64% [22] of patients and in 29.8% [50], 42% [51], 49% [52], and 67% [22] of family members of ICU patients. We hypothesized that patient PTSD rate will be 40% in the control group and 26% in the intervention group. To detect such a difference between the two groups with a type 1 error of 0.05 and a power of 80%, it is necessary to interview 352 patients (176 in each group) at 3 months. At 3 months, considering a mortality rate of 40% and a cumulative rate of 50% of re-hospitalization or impossibility of interviewing the patient (refusal, sequelae or impossibility to join them), we will include 700 patients and their family member in the 35 centers. In each center, 20 patients will be included (10 in the intervention arm, i.e., with an ICU diary, and 10 in the control arm).

### Statistical analysis

#### Outcome analysis

The analysis will be done according to the intention-to-treat principle. In each randomization group, we will report summary statistics according to the data (median and interquartile range, percentage with a 95% confidence interval).

The quantitative data will describe the patient’s characteristics and the results of the psychological impact using the IES-R and HADS scales in the two arms. The impact of ICU diaries on PTSD will be assessed using a Mantel-Haenszel chi-square test.

A Kruskal-Wallis test will be used to compare quantitative secondary endpoint.

In case of disequilibrium of patient characteristics between groups, further multivariate analyses will be computed.

**Fig. 2:**
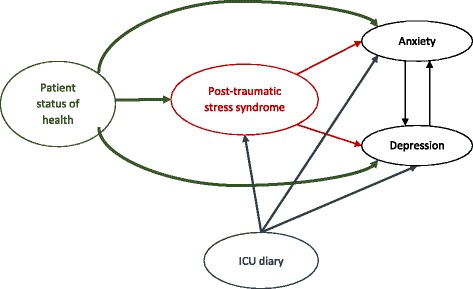
Example of a path diagram

To explain the effect of patient state of health, family characteristics and patient PTSD on both anxiety and depression, an exploration of direct and indirect effect of each variable on secondary outcomes will be performed using mediation analysis based on a structural equation model [53]. This approach consists of distinguishing measured variables which are indicators of unmeasured (latent) variables and exogenous variables which are exposure factors. Two steps will be performed: (1) an explanatory analysis to identify the latent variables and their measures and (2) the confirmatory analysis to assess direct or indirect relations between each latent variable. This second step is based on a conceptual model which clearly explains the relations between latent variables and their measures and defines the pathway between latent variables (Fig. 2). Because PTSD, anxiety, and depression will all be measured by a score, this approach will be more relevant to consider the different explicative factors. PTSD will be introduced as a latent variable to explain anxiety and depression [54, 55]. Finally, the center effect could be considered by introducing a hierarchical level in the mediation analysis. This point will be explored during the exploratory analysis.

All the analyses will be stratified by center. Data will be analyzed with the software SAS 9.4 (SAS Institute Inc., Cary, NC, USA). The results will be reported using the Consolidated Standards of Reporting Trials (CONSORT) criteria.

Figure 2 reports an example of a path diagram. Five latent variables are considered: (1) patient status of health, measured by ICU admission variables; this is supposed to impact the PTSD, but also anxiety and depression; (2) the ICU diary is considered here as a latent variable which can impact PTSD, anxiety and depression; (3) PTSD can have a direct effect on anxiety and depression; (4) anxiety, and (5) depression are the outcome and are considered inter-dependent. The model allows assessing direct effect between latent variables but also indirect effect, such as the effect of the ICU diary on anxiety which is mediated by PTSD.

### Quantitative diary content analysis

The content of a random sample of the study diaries will be analyzed with a grid (Table 1) already published [22]. This grid was build with a panel comprising 11 members (three ICU physicians including two from other units, one ICU nurse, two psychologists, two hospital visiting volunteers, one person from the general population with a history of admission to another ICU, one former patient of our ICU, and his wife) using a Delphi technique. Six categories were defined and will serve for the analysis of the content of the diaries. We will report quantitatively the categories and themes which are represented most often in the diaries and will give examples of verbatim narratives for each of them.

### Qualitative analysis of patient interviews

These interviews will be conducted in the intervention arm and will describe how the patient uses their diary. CV will conduct the patient interview by making direct phone calls. Each interview will be audio-recorded, transcripted verbatim, and qualitatively analyzed to capture the subjective use of the diary by the patient. This qualitative analysis will be done by CF and LF. A large sample of interviews will be subjected to the general inductive approach [56] while the others will be subjected to an interpretative phenomenological analysis [57] in order to understand in depth the meaning-making processes of the patients requiring intensive care.

### Study management

The Steering Committee comprises two intensivists (MGO, JFT), three psychologists (CF, LF, and CV) and a biostatistician (SR). Three clinical research assistants completed the team. MGO visited all the centers and has trained the teams to write in the diaries. MGO and JFT are in charge of addressing any questions from the investigators and for checking inconsistencies. SR is in charge of the website and will analyze the quantitative data with JFT. CV will perform the interview with the patients and their families at 3 months, blind to the arm of randomization. CF and LF will be in charge of the qualitative analysis of the content of the diaries and of the interviews with the patients at 6 months. In each ICU, a local investigator (NAB, LA, JB, NB, EB, LB, CB, FC, BF, AG, XF, HG, RH, OH, MJ, EK, AL, OL, VM, EM, JPM, YM, EPD, AR, JPR, FS, GS, FT, NTR, MT, SV, and IV) participated in the reflection of the standardized ICU diary, is responsible for the continuous training of the new ICU staff hired during the study, and enrollment with inform consent of the patients and relatives. The local investigators are responsible of the acquisition of all study data and for sending all the study documents. Each local investigator is accountable for the accuracy in ensuring that any part of the work and related questions is appropriately resolved.

## Discussion

With the development of the technical ICU, the prevention of sequelae is an increasingly important health problem. Reducing the psychological consequences of the post-intensive syndrome is one of the major challenges for intensivists. This study should provide insights on the impact of the ICU diary on post-traumatic consequences for patients and for their families. This tool has no financial cost, except the time offered by the caregivers to patients and family for filling it in. Until now, the ICU diary has been used in some ICUs, particularly those focused on patient- and family centered care. If a positive effect of the diary is demonstrated, it could be included in programs of patient- and family centered care in France and in countries where ICU organization and healthcare worker to patient ratio is similar to that in France.

However, some issues related to this study design should be discussed. First, the fact that the intervention is not blinded for the healthcare workers should be considered in the results. Healthcare workers might provide more support to patients and families in the diary group because their relationship could be modified by the fact of giving more time and more attention to the patient and their family through the diary filling. However, the limitation of 10 diaries by center should limit this phenomenon, as each center should rarely have more than one patient at once with a diary. Although the results of the study will not enable us to differentiate what is important in a diary: the tool, the narrative or the relationship with the healthcare team. Nevertheless, we chose to perform a content analysis by two psychologists using a previously published grid. This will permit a qualitative description of the diaries content, which will contribute to explaining the results.

The fact that patients or families may mention their diary during the interview and reveal their randomization arm is another study limitation. However, such cases will be noticed in the flow chart of the study. In addition, this bias is limited by the fact that the interview will use questionnaires focusing only on anxiety, depression, and post-traumatic symptoms. The questionnaire about the memory recollection has not open questions but only questions with some suggested answers. So it is close question compared to open questions when the patient can freely speak.

In conclusion, the results of this study are expected to show a positive effect of an ICU diary written by healthcare workers and families on the wellbeing of patients and families in the post-ICU period.

## Trial status

This trial is ongoing.

## Additional files

Additional file 1: SPIRIT 2013 Checklist: recommended items to address in a clinical trial protocol and related documents^*^. (DOC 121 kb)

Additional file 2: World Health Organization Trial Registration Data Set^*^. (XLSX 10 kb)

Additional file 3: Participants’ centers. (DOCX 13 kb)

## Abbreviations

CCTIRS: Comité Consultatif sur les Traitements de l’Information en matière de Recherche dans le domaine de la Santé
CNIL: Commission Nationale de l’Informatique et des Libertés
CONSORT: Consolidated Standards of Reporting Trials
HADS: Hospital Anxiety and Depression Scale
ICU: Intensive care unit
IES-R: Impact of Event Scale-Revised
PICS: Post-intensive care syndrome
PICS-F: Post-intensive care syndrome-family
