# Supplementary material for: The ICU-Diary study: prospective, multicenter comparative study of the impact of an ICU diary on the wellbeing of patients and families in French ICUs
Source: Trials. 2017 Nov 15;18:542. doi: 10.1186/s13063-017-2283-y (PMC5688734; doi:10.1186/s13063-017-2283-y)
Supplement: Supplementary file 3 — Participants’ centers. (DOCX 13 kb) [file 13063_2017_2283_MOESM3_ESM.docx]

Participants centers

| 1. Dr Sandrine Valade, Medical ICU, Saint Louis University Hospital, Paris |
| --- |
| 2. Dr Marina Thirion, Medical-Surgical ICU, General Hospital Victor Dupouy, Argenteuil |
| 3. Dr Xavier Forceville, Medical-Surgical ICU, Est Francilien Hospital Network, Meaux |
| 4. Dr Rebecca Halidfar, Medical ICU, Albert Michallon University Hospital, Grenoble |
| 5. Dr Isabelle Vinatier, Medical-Surgical ICU, Les Oudaries Hospital, La Roche-sur-Yon |
| 6. Dr Virginie Maxime, Medical ICU, Raymond Poincaré University Hospital, Garches |
| 7. Pr Jean Paul Mira, Medical ICU, Cochin University Hospital, Paris |
| 8. Dr Naike Bige, Medical ICU, Saint Antoine University Hospital, Paris |
| 9. Pr Laurent Argaud, Medical ICU, Edouard Herriot University Hospital, Lyon |
| 10. Dr Amélie Bazire, Medical ICU, la cavale blanche University Hospital, Brest |
| 11. Dr Jean-Philippe Rigaud, Medical-Surgical ICU, General Hôpital, Dieppe |
| 12. Dr Nathalie Thieulot-Rolin, Medical-Surgical ICU, General Hôpital, Melun |
| 13. Dr Paul-Henri Jost, Medical-Surgical ICU, Henri Mondor University Hospital, Créteil |
| 14. Dr Emmanuelle Mercier, Medical-Surgical ICU, Bretonneau University Hospital, Tours |
| 15. Dr Hubert Grand, Medical-Surgical ICU, General Hospital, Libourne |
| 16. Dr Marie Thuong, Medical-Surgical ICU, General Hospital Rene Dubos, Pontoise |
| 17. Dr Alain Gaffinel, Medical-Surgical ICU, Institut Gustave Roussy, Villejuif |
| 18. Pr Jean-Pierre Quenot, Medical ICU, Dijon Bourgogne University Hospital, Dijon |
| 19. Dr Eric Boulet, Medical-Surgical ICU, General Hospital Rene Dubos, Pontoise |
| 20. Dr Julio Badie, Medical-Surgical ICU, General Hospital Belfort-Montbeliard, Belfort |
| 21. Dr Yannick Monseau, Medical-Surgical ICU, General Hospital, Périgueux |
| 22. Dr Pierre Cougot, Medical-Surgical ICU, Rangueil University Hospital, Toulouse |
| 23. Pr Lila Bouadma, Medical ICU, Bichat University Hospital, Paris |
| 24. Dr Olivier Lesieur, Medical-Surgical ICU, General Hospital, la Rochelle |
| 25. Dr Lilia Soufir, Medical-Surgical ICU, Saint Joseph Network Hospital, Paris |
| 26. Pr Alexandre Lautrette, Medical ICU, Gabriel-Montpied University Hospital, Clermont Ferrand |
| 27. Dr Bernard Floccard, Medical ICU, Edouard Herriot University Hospital Lyon |
| 28. Dr Georges Simon, Medical-Surgical ICU, General Hospital, Troyes |
| 29. Pr Fabienne Tamion, Medical ICU, Andre Nicolle University Hospital, Rouen |
| 30. Pr Mercé Jourdain, Group of medical ICUs, Calmette University Hospital, Lille |
| 31. Dr Olfa Hamzaoui, Medical ICU, Béclère University Hospital, Clamat |
| 32. Pr Eric Kipnis, Surgical ICU, Calmette University Hospital, Lille |
| 33. Dr Erika Parmentier, Group of medical ICUs, Calmette University Hospital, Lille |
| 34. Dr Cédric Bretonnière, Medical ICU, University Hospital, Nantes |
| 35. Dr François Santoli, Medical ICU, General Hospital Robert Ballanger, Aulnay-sous-bois |
